# Supplementary material for: Autologous Adipose-Derived Mesenchymal Stem Cells Combined with Shockwave Therapy Synergistically Ameliorates the Osteoarthritic Pathological Factors in Knee Joint
Source: Pharmaceuticals (Basel). 2021 Apr 1;14(4):318. doi: 10.3390/ph14040318 (PMC8065528; doi:10.3390/ph14040318)
Supplement: Supplementary file 1 [file pharmaceuticals-14-00318-s001.pdf]

Autologous adipose-derived mesenchymal stem cells combined with shockwave therapy synergistically ameliorates the osteo-arthritic pathological factors in knee joint

Jai-Hong Cheng[1,2,3*], Ke-Tien Yen[3], , Wen-Yi Chou[1,4], Shun-Wun Jhan[1,4], Shan-Ling Hsu[1,4,5], Jih-Yang Ko[1,4], Ching-Jen Wang[1,4], Chun-En Aurea Kuo[6], Szu-Ying Wu[6], Tsai-Chin Hsu[1,4] and Chieh-Cheng Hsu[1,4*]

[1]Center for Shockwave Medicine and Tissue Engineering, Kaohsiung Chang Gung Memorial Hospital and Chang Gung University College of Medicine, Kaohsiung 833, Taiwan.

[2]Medical Research, Kaohsiung Chang Gung Memorial Hospital and Chang Gung University College of Medicine, Kaohsiung 833, Taiwan.

[3]Department of Leisure and Sports Management, Cheng Shiu University, Kaohsiung 833, Taiwan.

[4]Department of Orthopedic Surgery, Sports Medicine, Kaohsiung Chang Gung Memorial Hospital and Chang Gung University College of Medicine, Kaohsiung 833, Taiwan.

[5]Fooyin University, School of Nursing, Kaohsiung 831, Taiwan.

[6]Department of Chinese Medicine, Kaohsiung Chang Gung Memorial Hospital and Chang Gung University College of Medicine, Kaohsiung, 833401, Taiwan

* Correspondence: Jai-Hong Cheng, Ph.D., Email: cjh1106@cgmh.org.tw. Tel.: +886-7-733-6422; Chieh-Cheng Hsu, MD, Email: t1234@cgmh.org.tw

Supplemental Figure 1

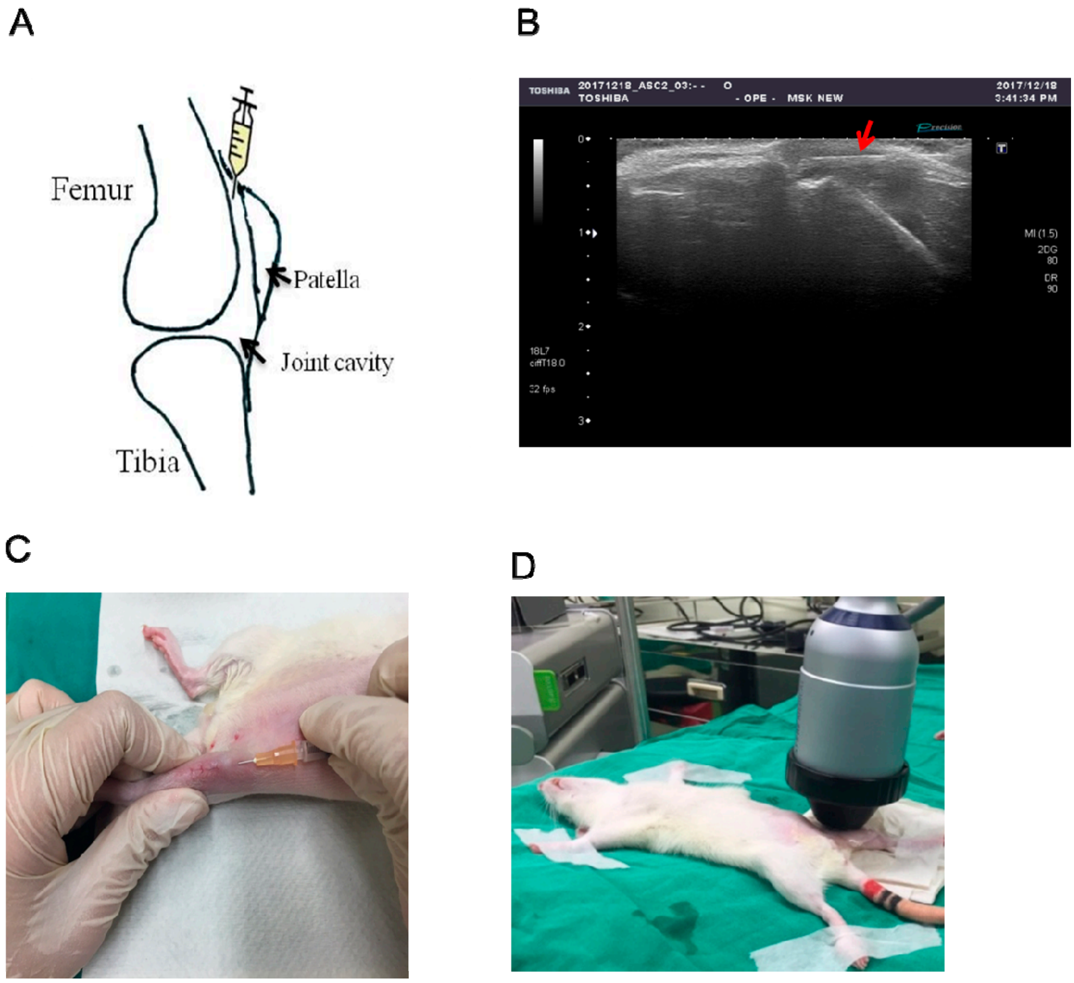

Supplemental Figure 1. The autologous adipose-derived mesenchymal stem cells (ADSCs) injection and shockwave (SW) therapy. (A) The cartoon is illustrated the autologous ADSCs injection into left rat knee above the patella. (B) The ultrasound is used to guide the injection of autologous ADSCs into rat knee. The needle is indicated as red arrow in the joint cavity. (C) The image is displayed that autologous ADSCs are injecting into rat knee. (D) After 30 min injection of autologous ADSCs, the SW is applying on the medial rat knee. N = 8.

Supplemental Table 1. OARSI analysis after treatments.

OARSI Analysis	Sham	OA	SW	ADSC1	ADSC2	ADSC1+SW	ADSC2+SW
grade	0.00±0.00	4.96±0.12	2.88±0.22	4.71±0.10	4.13±0.21	3.42±0.08	1.92±0.31
stage	0.00±0.00	2.67±0.21	2.50±0.22	2.67±0.21	2.17±0.17	2.17±0.17	0.67±0.21
Score=grade × stage	0.00±0.00	13.29±1.24	7.33±1.09	12.63±1.16	9.08±1.21	7.38±0.50	1.50±0.53
